# The role of short-chain fatty acids in inflammatory skin diseases

**DOI:** 10.3389/fmicb.2022.1083432

**Published:** 2023-02-02

**Authors:** Xianjun Xiao, Xiaoshen Hu, Junpeng Yao, Wei Cao, Zihao Zou, Lu Wang, Haiyan Qin, Dongling Zhong, Yuxi Li, Peiwen Xue, Rongjiang Jin, Ying Li, Yunzhou Shi, Juan Li

**Affiliations:** ^1^College of Health Preservation and Rehabilitation, Chengdu University of Traditional Chinese Medicine, Chengdu, Sichuan, China; ^2^College of Acupuncture and Tuina, Chengdu University of Traditional Chinese Medicine, Chengdu, Sichuan, China

**Keywords:** short-chain fatty acid (SCFA), inflammatory skin disease, histone deacetylase (HDAC), atopic dermatitis, urticaria, psoriasis, eczema, acne

## Abstract

Short-chain fatty acids (SCFAs) are metabolites of gut microbes that can modulate the host inflammatory response, and contribute to health and homeostasis. Since the introduction of the gut-skin axis concept, the link between SCFAs and inflammatory skin diseases has attracted considerable attention. In this review, we have summarized the literature on the role of SCFAs in skin inflammation, and the correlation between SCFAs and inflammatory skin diseases, especially atopic dermatitis, urticaria, and psoriasis. Studies show that SCFAs are signaling factors in the gut-skin axis and can alleviate skin inflammation. The information presented in this review provides new insights into the molecular mechanisms driving gut-skin axis regulation, along with possible pathways that can be targeted for the treatment and prevention of inflammatory skin diseases.

## 1. Introduction

Inflammatory skin disease refers to the inflammation response in the skin, which manifests as skin redness, swelling, itching or scaling. Common inflammatory skin diseases, including atopic dermatitis (AD), psoriasis, acne, and urticaria, are associated with other comorbidities and impose a significant burden on the patients (Narla and Silverberg, 2020). Although the above diseases vary in their clinical presentation, they may share common physio-pathological pathways (Diotallevi et al., 2022). Recent studies have shown that the gut microbiome is an important factor influencing host immunity, inflammation and metabolism (Yao et al., 2022). The gut-skin axis is a fairly recent concept that refers to the bidirectional relationship between the gut microbiome and skin, and an increasing body of evidence suggests that changes in the gut microbiome is related with skin inflammation (Kim et al., 2020; Chun et al., 2021). The gut and the skin are both highly innervated and vascularized organs, and harbor numerous resident microorganisms with similar functions (O’Neill et al., 2016). Furthermore, various skin conditions have been linked to an altered gut microbiome (Pessemier et al., 2021).

Short-chain fatty acids (SCFAs), mainly including acetate, propionate, butyrate, isobutyrate, valerate, and isovalerate, contain less than 6 carbon atoms and are the final product of the fermentation of resistant starch and dietary fiber by specific gut microbiota (Tan et al., 2014; Rauf et al., 2022). SCFAs are transported from the intestine to distant organs and tissues through the peripheral circulation (Canfora et al., 2015; Hee and Wells, 2021), and bind to G protein-coupled receptors (GPCRs) that are expressed on skin cells, leukocytes, neutrophils, and other types of cells, thereby exerting direct influence on tissue metabolism and function (Krejner et al., 2018; Schlatterer et al., 2021). Recent studies have shown that SCFAs mitigate inflammation by regulating the production of cytokines by immune cells such as neutrophils, macrophages, dendritic cells (DCs) and T-cells (Yao et al., 2022). However, the role of SCFAs in inflammatory skin diseases has not been completely elucidated. In this review, we summarized the literature on the relationship between SCFAs and skin inflammation, and discussed the therapeutic potential of SCFAs against inflammatory skin diseases.

## 2. The potential pathway of SCFAs in inflammatory skin diseases

**FIGURE 1:**
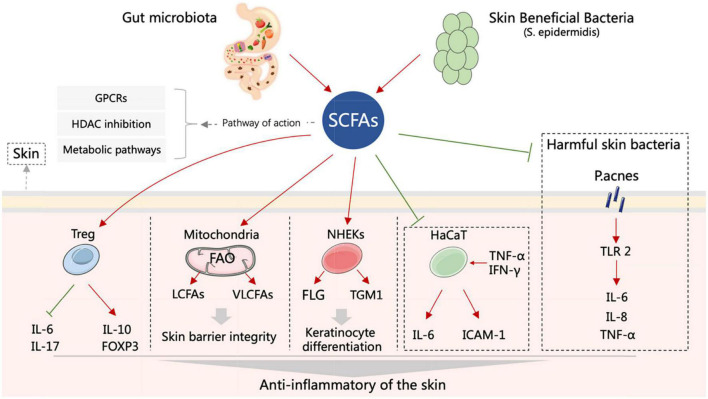
Schematic representation of the roles of SCFAs in inflammatory skin diseases. Both gut and skin microbiota can produce SCFAs. SCFAs can enhance the activity of Treg, improve mitochondrial metabolism, promote keratinocyte differentiation, reduce the expression of inflammatory factors in HaCaT cells, and inhibit the inflammatory response induced by *P. acnes* that results in skin inflammation relief and skin barrier improvement. FLG, filaggrin; FOXP3, forkhead box protein 3; GPCRs, G protein-coupled receptors; HaCaT, human immortalized keratinocytes; ICAM-1, intercellular adhesion molecule-1; IFN-γ, interferon-γ; IL-6, interleukin-6; IL-8, interleukin-8; IL-10, interleukin-10; IL-17, interleukin-17; LCFAs, long-chain fatty acids; NHEKs, normal human epidermal keratinocytes; *P. acnes*, *Propionibacterium acnes*; *S. epidermidis*, *Staphylococcus epidermidis*; SCFAs, short chain fatty acids; TGM1, transglutaminase-1; TLR-2, toll-like receptor-2; TNF-α, tumor necrosis factor-α; Treg, regulatory T-cells; VLCFAs, very long-chain fatty acids.

The potential anti-inflammatory mechanisms of SCFAs in inflammatory skin diseases are related to specific membrane receptors, histone deacetylase (HDAC) inhibitors, and metabolic pathways (Figure 1).

### 2.1. SCFA receptors pathway

Short-chain fatty acid receptors are activated upon binding to the ligand and facilitate SCFAs entry into cells (Thiruvengadam et al., 2021). The established SCFAs receptors include G protein-coupled receptor 41 (GPR41) [a.k.a free fatty acid receptor 3 (FFAR3)] (Deng et al., 2022), G protein-coupled receptor 43 (GPR43) [a.k.a. free fatty acid receptor 2 (FFAR2)] (Schlatterer et al., 2021), G protein-coupled receptor 109A (GPR109A) [a.k.a. hydroxycarboxylic acid receptor 2 (HCA2)] (Krejner et al., 2018), Olfr-78 (for murine), OR51E2 (for human) (Ohira et al., 2017), aryl-hydrocarbon receptor (AHR) (Rosser et al., 2020) and peroxisome proliferator-activated receptors γ (PPARγ) (Aguilar et al., 2018; Yao et al., 2022). SCFA-mediated receptors play an important role in regulating the inflammatory response of the host. FFAR2-deficient mice exhibit exacerbated inflammation in mouse models of arthritis, colitis, and asthma, and similar dysregulation in germ-free mice with low or no SCFA expression (Maslowski et al., 2009). These results show that SCFAs interact with GPR43 profoundly affect the inflammatory response (Maslowski et al., 2009). SCFAs act via FFAR2/3 to attenuate the secretion of various inflammatory cytokine and modulate the inflammatory response of human monocytes (Ang et al., 2016). Moreover, SCFAs activate GPR43 and GPR109a to regulate inflammatory response of the host by downregulation of NF-κB signaling pathway (Singh et al., 2014; Cleophas et al., 2016). Additionally, the PPARγ not only competitively inhibits the activation of NF-κB (Mao et al., 2012), but also cooperates with SCFAs to suppress the phosphorylation of NF-κB signaling pathway (Zhang et al., 2022).

Recent studies have shown that SCFA receptors are closely related to the pathogenesis of inflammatory skin diseases. AHR expression significantly increase in the serum and skin lesions of patients with AD (Kim et al., 2014; Beránek et al., 2018; Hu et al., 2020), whereas PPARγ, GPR43, and GPR109a downregulate in psoriasis (Lin et al., 2022). Moreover, a study has indicated that butyrate can increase the expression of GPR43 and GPR109a in psoriasis and exert anti-inflammatory effects (Krejner et al., 2018). The binding of these receptors to SCFAs activates intracellular signaling pathways that regulate cellular responses, immune function, and inflammation (Marinissen and Gutkind, 2001; Trompette et al., 2014; Haase et al., 2018).

Taken together, alterations in the expression of SCFAs-mediated receptors may involve in the pathogenesis of inflammatory skin diseases (Krejner et al., 2018). Existing evidence suggests that SCFAs may play an anti-inflammatory role in inflammatory skin diseases by restoring the expression of SCFAs-mediated receptors, but the specific mechanism is rarely reported. Future studies are needed to gain insight into the anti-inflammatory role of SCFAs in inflammatory skin diseases.

### 2.2. HDAC inhibition and associated pathway

The dynamic balance between histone acetylases (HATs) and HDACs controls chromatin structure and gene expression (Koh et al., 2016). HDAC inhibitors deacetylate histones in the promoter regions, resulting in the transcriptional activation of the downstream genes. The anti-inflammatory effects of SCFAs have been attributed to their ability to inhibit HDAC activity. For instance, SCFA blocks *CXCL10* release through HDAC inhibition, reduce inflammation and maintain immune homeostasis and gut health (Korsten et al., 2022). Furthermore, butyrate promotes *FOXP3* expression in naive CD4^+^ T-cells and induces their differentiation into peripheral-derived Tregs (pTregs) by inhibiting HDACs (Martin-Gallausiaux et al., 2018), while acetate, propionate and butyrate can modulate the immune response of DCs, macrophages and Treg cells by inhibiting HDACs (Thiruvengadam et al., 2021). Imiquimod (IMQ)-induced psoriasis-like skin inflammation model reduces the suppressive activity of Treg, and then upregulates IL-17 and IL-6, and downregulates IL-10 and FOXP3, whereas butyrate can reverse these progressions by inhibiting HDACs (Schwarz et al., 2021) (Figure 1). In LPS-activated neutrophils, application of both butyrate and propionate inhibits NF-κB activity and reduces TNF-α production mainly by inhibiting HDACs (Aoyama et al., 2010). In addition, SCFAs can alleviate systemic inflammation by significantly reducing the production of TNF-α and IL-6 through the downregulation of HDACs mRNAs (Eslick et al., 2022). *Propionibacterium acnes* in the skin activate toll-like receptor-2 (TLR-2) and promote the secretion of inflammatory factors such as IL-6, IL-8, and TNF-α, thereby inducing skin inflammation. This process may be attenuated by SCFAs via inhibiting HDACs (Wang et al., 2016). Furthermore, Butyrate enhances the expression of filaggrin (FLG) and transglutaminase-1 (TGM1) and promotes normal human epidermal keratinocytes (NHEKs) terminal differentiation to maintain skin homeostasis by inhibiting HDACs (Carrion et al., 2014) (Figure 1).

Overall, SCFAs can reduce the release of inflammatory cytokines and promote the differentiation of epidermal keratinocytes via inhibiting HDACs, thereby playing an anti-inflammatory role in inflammatory skin diseases.

### 2.3. Metabolic pathway

Short-chain fatty acids can affect cellular metabolism by promoting mitochondrial fatty acid β-oxidation (FAO) (Trompette et al., 2018; Bachem et al., 2019). Skin barrier dysfunction is a common pathological feature of inflammatory skin diseases (Diotallevi et al., 2022; Trompette et al., 2022). Recent studies have shown that SCFAs is able to improve the skin barrier and relieve skin inflammation by altering mitochondrial metabolism and function (Trompette et al., 2022). Butyrate is metabolized by epidermal keratinocytes, which in turn enhances the synthesis of keratinocyte-derived long-chain fatty acids (LCFAs) and very long-chain fatty acids (VLCFAs), a key event in the subsequent generation of ceramides that are critical to skin barrier function. Interestingly, this phenomenon is limited to the skin, since butyrate has little effect on systemic LCFA and VLCFA levels. Furthermore, butyrate can also enhance LCFA uptake (Bachem et al., 2019; Trompette et al., 2022) (Figure 1). Genetic variation in the mitochondrial genome is associated with chronic inflammatory skin disease. Intriguingly, in mice with antibody-induced dermatitis, propionate-treated disease progression pattern is similar to that of mitochondrial gene variant (B6-mt^FVB^) mice, both effectively reducing the severity of inflammatory skin disease (Schilf et al., 2021).

Thus, the anti-inflammatory effects of butyrate on the skin can be attributed to fueling FAO (directly and indirectly) and altering mitochondrial metabolism, promoting keratinocyte differentiation, and improving the skin barrier.

## 3. Relationship between SCFAs and inflammatory skin diseases

Many studies have reported the interaction between SCFAs and inflammatory skin diseases. We reviewed the effects of SCFAs on AD, eczema, acne, chronic spontaneous urticaria (CSU), and psoriasis.

### 3.1. SCFAs in atopic dermatitis

Atopic dermatitis is a chronic inflammatory skin disease that is caused by a number of genetic, environmental, and immunological factors (Williams and Flohr, 2006). Recent studies show that the symptoms of AD are the result of a systemic immune response induced by changes in the gut microecology (Kim et al., 2020). Furthermore, evidence demonstrates that the intestinal levels of SCFAs correlate with the onset of AD. For instance, Patients with AD have low levels of fecal SCFAs, which corresponds to a significant reduction in the abundance of SCFA-producing bacteria (Reddel et al., 2019). Similar trends have been observed in animal models as well (Kim et al., 2019). Moreover, the severity of AD correlates negatively with the abundance of butyrate-producing bacteria (Nylund et al., 2015). A follow-up study of children aged 6–24 months revealed lower levels of butyrate and valerate in infants with transient AD compared to the healthy infants and those with persistent AD (Park et al., 2020). Other studies have shown that infants younger than 3 years with AD have lower amounts of propionate and butyrate in their first year (Ta et al., 2020). Similar patterns have been observed in mixed age groups (Song et al., 2016) (see Table 1). Consistent with the above findings, mice exhibiting AD-like symptoms induced with transdermal injection of 2,4-dinitrochlorobenzene have lower SCFA levels compared to the healthy littermates. Furthermore, the symptoms of AD in these mice are relieved after treatment, and the SCFA levels are normalized (Chun et al., 2021). These findings underscore the role of the SCFAs as signaling molecules of the gut-skin axis.

**TABLE 1:** Expression levels of SCFAs in different inflammatory skin diseases.

Disease	Age	SCFAs involved							Samples	Expression levels (compared to normal)	Association with disease	Ref.
		C2	C3	C4	iC4	C5	iC5	iC6				
AD	0∼3 Y	√	√	√		√			Feces	C4↓, C5↓		Park et al., 2020
	3∼12 M	√	√	√					Feces	C2↓, C3↓, C4↓	The reduction in butyrate and propionate levels was significantly associated with AD.	Ta et al., 2020
		√	√	√					Feces	C3↓, C4↓		Song et al., 2016
Eczema	Infant	√	√	√		√			Feces	C4↓, C5↓	Butyrate and valerate levels were negatively correlated with SCORAD at 12 months of age.	Kang M. J. et al., 2021
	Infants	√	√	√	√				Feces	C3↑, C4↑ at 12 weeks. This pattern was reversed at 26 weeks with C3↓ and C4↓	Butyrate and valerate levels were negatively correlated with eczema.	Wopereis et al., 2018
	Infants	√	√	√					Feces	C2↓, C4↓		Kim H. K. et al., 2015
	Children	√	√	√	√	√	√	√	Feces	C5↓	The level of fecal valerate at 1 year of age was inversely associated with eczema at 13 years of age.	Batta et al., 2021
	Children	√	√	√	√	√			Feces		The expression level of valerate was negatively correlated with the incidence rate of eczema.	Gio-Batta et al., 2020
CSU	12∼60 Y				√				Feces	iC4↓		Wang et al., 2021
	18∼75 Y			√					Feces and blood	C4 metabolism↓	Lower butyrate levels due to dysregulation of the gut microbiota may play an important role in the pathogenesis of CSU.	Wang et al., 2020
Psoriasis		√	√		√	√			Blood	iC4↑, C5↑, C2↓, C3↓		Khyshiktuev et al., 2008

AD, atopic dermatitis; C2, acetate; C3, propionate; C4, butyrate; i-C4, iso-butyrate; C5, valerate; i-C5, iso-valerate; i-C6, iso-caproate; CSU, chronic spontaneous urticaria; HDACi, histone deacetylases inhibition; M, months old; Y, years old; Ref., references; SCORAD, scoring of atopic dermatitis.

The development of AD is closely related to immune dysregulation, microbial exposure, disrupted skin barrier and intestinal dysbiosis (Elias and Steinhoff, 2008). An imbalance between the pro-inflammatory CD4^+^IL17^+^ T-cells and the anti-inflammatory CD4^+^FOXP3^+^ regulatory T-cells (Tregs) in the gut may be a key determinant of early AD development (Kim et al., 2020). Both propionate and butyrate contribute to the accumulation of Tregs in the colon through activation of DCs and T-cells or G protein signaling (Arpaia et al., 2013; Smith et al., 2013). Therefore, restoring the balance between the intestinal T-cell subpopulations by SCFAs supplementation may mitigate the symptoms of AD (Kim et al., 2020). Low levels of SCFAs may also increase intestinal permeability and disrupt its barrier function, eventually triggering AD (Benedetto et al., 2011). SCFAs are partly absorbed by the intestine and the remainder enter the bloodstream, and are distributed to various organs and tissues (Boets et al., 2017; Vonk and Reckman, 2017). Therefore, SCFAs may act directly on skin cells to regulate the barrier function (Schauber et al., 2006, 2008; Sunkara et al., 2012; Carrion et al., 2014; Wei et al., 2017). Keratinocytes exposed to TNF-α and IFN-γ express aberrantly high levels of the pro-inflammatory cytokine IL-6, and the intercellular adhesion molecule-1 (ICAM-1), which in turn recruit monocytes to the epidermis. These inflammatory keratinocytes involve in the pathophysiology of AD. SFCAs markedly reduce *IL-6* and *ICAM-1* mRNA levels in human keratinocytes (HaCaT cells) stimulated with TNF-α and INF-γ *in vitro* (Chun et al., 2021) (Figure 1). In addition, butyrate also inhibits the inflammatory phenotype of cultured human keratinocytes by inducing acetylation of histone H3 lysine 9 (AcH3K9) (Meijer et al., 2010; Traisaeng et al., 2019).

The pathogenesis of AD is also related to an imbalance in the skin microecology. The skin of AD patients is more susceptible to the colonization and overgrowth of pathogenic bacteria due to the lower abundance of probiotic bacteria (Leung, 2013), which inhibit the growth of pathogens through metabolites (Iwase et al., 2010; Naik et al., 2012; Ren et al., 2013). For instance, the SCFAs produced during fermentation by beneficial bacteria have an inhibitory effect on community-associated methicillin-resistant *Staphylococcus aureus* (Kao et al., 2017). *S. aureus* is abundant in the AD skin, and promotes inflammation by binding to and activating TLR-2, resulting in IL-4-mediated inhibition of IL-10 (Leung, 2013; Kaesler et al., 2014). SCFAs not only improve the local skin microflora but also restrain the migration of immune cells and the production of TNF-α, IL-6, and IL-8 (Wang et al., 2016) (Figure 1).

In summary, SCFAs are closely related to the pathogenesis of AD. The level of SCFAs in AD patients is generally lower than that in healthy controls. Low levels of fecal SCFAs may increase intestinal permeability and induce AD. However, transcutaneously induced AD-like animal models can also reduce intestinal SCFAs levels, but little is known about SCFAs expression in lesion skin. In future studies, it is recommended to add the measurement of SCFAs expression in AD-damaged skin to better illustrate the role of SCFAs in inflammatory skin diseases.

### 3.2. SCFAs in eczema

Eczema is a common inflammatory skin disease with underlying genetic and environmental causes. Studies show that gut microecology is an important environmental factor in the occurrence of eczema, and SCFA levels can predict the risk of eczema (Kim H. K. et al., 2015). The decrease in SCFA level precedes eczema symptoms (Sandin et al., 2009; Kim H. K. et al., 2015). Lower levels of fecal butyrate and valerate have been observed in 6-month-old infants with eczema, and butyrate levels, in particular, have negative correlation with the development of eczema (Wopereis et al., 2018; Kang M. J. et al., 2021). This is consistent with the inverse correlation between low microbial complexity in early infancy and the likelihood of allergies in later life (Wang et al., 2007; Bisgaard et al., 2011; Ismail et al., 2012; Victora et al., 2016; Roduit et al., 2019; Cheng et al., 2022). Furthermore, most patients with eczema have low levels of SCFAs, and low fecal valerate levels in childhood is related to a higher incidence of eczema in youth (Gio-Batta et al., 2020; Batta et al., 2021). These findings indicate that low levels of SCFAs may increase the susceptibility to eczema. However, research concerning the role of SCFAs in the pathogenesis of eczema is mainly focused on infants and young children, and it is unclear whether these findings can be extrapolated to adult patients as well. More clinical evidence is needed to elucidate the correlation between SCFAs and eczema.

Interleukin-17 (IL-17) is expressed in different subtypes of eczema and can enhance the inflammatory symptoms (Simon et al., 2014). Valerate can inhibit IL-17 release by promoting IL-10 production via the CD4^+^ T-cells and regulatory B-cells, a process which involves reprogramming lymphocyte metabolism and inhibition of HDAC activity (Yuille et al., 2018; Luu et al., 2019). Butyrate can also diffuse through the intestinal epithelial cells, promote the differentiation of naive T-cells to Tregs, and prevent allergic diseases such as eczema (Kim H. K. et al., 2015). Furthermore, the development of eczema is often associated with impaired skin barrier function (Lee et al., 2021). Keratin (KRT1) and FLG play a crucial role in the maintenance of a healthy skin barrier (Freedberg et al., 2001; Pfisterer et al., 2021). KRT1-deficient mice have a compromised skin barrier, and their skin transcriptome resembles that of eczema skin (Roth et al., 2012). Valerate can improve skin tissue integrity and barrier function by upregulating KRT1 and tight junction proteins (Li et al., 2020; Nguyen et al., 2020). Recent studies reveal that the etiology of eczema is closely related to the genetic loss of FLG, which leads to dryness and impaired skin barrier function (Kalb et al., 2022). Butyrate and propionate can increase the expression of FLG protein by inhibiting the activity of HDAC, and restoring the function and permeability of the epidermal barrier (Kleuskens et al., 2022).

Taken together, low levels of SCFAs may increase susceptibility to eczema. SCFAs are capable of improving the skin barrier and alleviating the skin inflammation of eczema. However, the clinical application of SCFA in eczema needs further investigation.

### 3.3. SCFAs in acne

Acne is a common inflammatory skin condition caused by the overgrowth of *P. acnes* (Kong and Segre, 2012; Scanlan et al., 2012; Wu et al., 2021). The probiotic *Staphylococcus epidermidis* can inhibit bacterial colonization and *P. acnes*-induced inflammation at the lesions by producing SCFAs (Wang et al., 2016). Propionate and butyrate are ligands of GPR41 (Park et al., 2007), which are upregulated through the SCFAs produced by *S. epidermidis*. In addition, the latter may alleviate *P. acnes*-induced inflammation by inhibiting HDAC activity (Wang et al., 2016). The protective function of SCFAs depends on the routes of administration. Subcutaneous injection of SCFAs and *P. acnes* in mice abrogated the upregulation of inflammatory genes, as opposed to topical application of the SCFAs on the back (Sanford et al., 2016). These results indicate that SCFAs can inhibit the growth of *P. acnes*, and their anti-inflammatory effects depend on the mode of application. The exact mechanisms underlying the differential effects of SCFAs on the epidermal and subcutaneous tissues are unclear. The same SCFAs may exert different effects in different application environments. How to apply SCFAs safely and effectively against skin inflammation (the dose of SCFAs, the site, and the application environment, etc.) become research highlights.

### 3.4. SCFAs in chronic spontaneous urticaria

Chronic spontaneous urticaria is a type of chronic inflammatory dermatosis that is predominantly mast cell-driven (Zuberbier et al., 2021; Peng et al., 2022). Alterations in gut metabolites may exacerbate the inflammatory response and immune dysfunction during the pathogenesis of CSU (Wang et al., 2020). In addition, reduced SCFAs accumulation due to an imbalance in the intestinal flora may play an important role in the pathogenesis of CSU (Wang et al., 2020). Clinical studies have shown that the levels of fecal isobutyrate (Wang et al., 2021) and serum butyrate (Wang et al., 2020) are lower in CSU patients compared to that in healthy individuals. This is consistent with the observation that butyrate represses mast cell proliferation, degranulation and cytokine production in mice (Galli et al., 1982; Diakos et al., 2006), and reduces the levels of inflammatory cytokines (Wang et al., 2018). SCFAs bind to receptors (such as GPR43 and PPARγ) that are expressed on mast cells (Sugiyama et al., 2000; Karaki et al., 2006), and inhibit the inflammatory response, mast cell maturation, and even dermatitis (Tachibana et al., 2008; Maslowski et al., 2009; Sina et al., 2009). Thus, SCFA-specific receptors expressed on mast cells are potential therapeutic targets for CSU. A recent study demonstrates that SCFAs suppress the activity of human and murine primary mast cells by inhibiting HDAC independent of GPR41, GPR43, and nuclear PPAR receptors (Folkerts et al., 2020). This suggests that SCFAs inhibit mast cell activation and inflammatory responses through multiple pathways. In addition, SCFAs can inhibit mast cell activation *in vitro* and *in vivo* at levels comparable to that in the intestine and serum of human, without affecting cell viability (Cummings et al., 1987; Wong et al., 2006). Therefore, it may be a promising direction to study the pathogenesis and treatment of CSU based on SCFAs.

### 3.5. SCFAs in psoriasis

Psoriasis is an immune-mediated inflammatory skin disease with complex pathogenesis (Rodriguez et al., 2014; Rendon and Schäkel, 2019). It manifests as chronic inflammation and subsequent epidermal hyperplasia, resulting in silvery scales and thickening of the skin (Rodriguez et al., 2014). Altered intestinal microecology is a pathological factor in psoriatic development (Lu et al., 2021). One study discovers that serum acetate and propionate levels are lower in patients with psoriasis compared to healthy individuals (Khyshiktuev et al., 2008). Furthermore, serum acetate and propionate levels correlate negatively with that of IL-23/IL-27 in an IMQ-induced mouse model of psoriasis, and administration of these SCFAs alleviates the inflammatory symptoms (Lu et al., 2021). Psoriatic progression depends on the activation of the TNF-α/IL-23/IL-17 signaling axis, and the hyperproliferation and aberrant differentiation of epidermal keratinocytes (Takeshita et al., 2017; Furue et al., 2018). IL-23 induces the transformation of Tregs to the T helper type 17 (Th17) cells, whereas IL-17A reduces transforming growth factor (TGF)-β1 production and Foxp3 expression, and inhibits Treg activity (Rodriguez et al., 2014; Stockenhuber et al., 2018; Kanda et al., 2021). Decreased Treg activity in patients with psoriasis is closely related to the level of SCFAs (Kanda et al., 2021), and the beneficial effects of SCFAs are also dependent on the role of Tregs (Schwarz et al., 2021). In addition, the genes involved in SCFA metabolism are significantly downregulated in patients with psoriasis, which may be associated with Treg dysfunction (Ahn et al., 2016). Administration of SCFAs can improve the symptoms of psoriasis by inhibiting HDACs in the Tregs and restoring their activity (Smith et al., 2013; Schwarz et al., 2021). Furthermore, SCFAs can promote the expansion of peripherally derived Tregs (Kaisar et al., 2017; Martin-Gallausiaux et al., 2018; Isobe et al., 2020), although there is a lack of psoriasis-targeted studies.

G protein-coupled receptor 109A and GPR43 are expressed at significantly lower levels in the keratinocytes of psoriasis patients compared to that of healthy individuals, and topical application of butyrate can restore their expression levels (Krejner et al., 2018). In addition, *in vitro* experiments have shown that butyrate can upregulate filaggrin and TGM1 transcripts in cultured human keratinocytes and promote the formation of cornified envelope (Carrion et al., 2014), which may help improve psoriatic lesions. In fact, topical application of SCFAs reduce both IMQ-induced psoriasis and systemic inflammatory responses in mice (Schwarz et al., 2021). Hence, these studies suggest that SCFAs may be involved in the regulation of psoriasis by restoring Treg activity and promoting the formation of cornified envelope.

## 4. SCFA-based therapy for inflammatory skin diseases

In recent years, SCFAs have emerged in the field of inflammatory skin diseases as an anti-inflammatory modulator, but it is still in its infancy. Therefore, understanding the application of SCFAs in the treatment of inflammatory skin diseases can pave a path for future clinical practice.

### 4.1. Topical administration of SCFAs in inflammatory skin diseases

The skin is the first line of defense against biological, physical, and chemical stresses (Coppola et al., 2022). Numerous viruses, bacteria, archaea, fungi, and mites reside on the skin surface which constitute the skin microbiome (Eisenstein, 2020). Skin-resident microbes maintain cutaneous homeostasis and regulate the local inflammatory response (Chen et al., 2018), and any disruption in the skin microbiome can induce an inflammatory reaction (Pessemier et al., 2021). Local application of SCFAs can promote the growth of beneficial microbes, and topical administration of probiotics promotes the release of SCFAs to mitigate skin inflammation (Kim C. H. et al., 2015). In a trial to validate acetate for AD, topical application of apple cider vinegar (0.5% acetate) for 2 weeks did not affect the skin microbiome of healthy subjects and AD patients compared to the water placebo group, and also did not prevent the colonization of *S. aureus* on the inflamed skin (Luu et al., 2021). However, no significant difference was observed in the diversity of the skin microbiome between the AD patients and healthy subjects at baseline (Luu et al., 2021). Therefore, the inhibitory effect of apple cider vinegar on *S. aureus* growth may depend on the bacterial strain and the levels of acetate (Fraise et al., 2013). On the other hand, in mice with IMQ-induced skin inflammation, topical injection of acetate led to further exacerbation of skin inflammation (Nadeem et al., 2017). Similarly, topically (2% w/w) and systemically administered (200 mmol/L) acetate was used to enhance psoriasis-like signs in an animal model of IMQ-induced psoriasis (Karamani et al., 2021). Therefore, the net effects of the actions of SCFAs are context-dependent and can be pro- or anti-inflammatory (Schilf et al., 2021). The exact effects of SCFAs may depend on the type of SCFAs, the delivery method, the levels and the health condition of hosts (Xiong et al., 2022). Moreover, fermentation initiators such as sucrose can selectively promote SCFA production by the probiotic *S. epidermidis*, and improve acne dysbiosis (Wang et al., 2016). Topical application of butyrate not only inhibits allergic contact inflammation in a mouse model (Schwarz et al., 2017), but also alleviates skin inflammation and the expression of inflammatory factors related to psoriasis (Schwarz et al., 2021).

However, most SCFAs have an offensive odor and are therapeutically effective only at certain concentrations (Kao et al., 2017; Traisaeng et al., 2019), which significantly limit their clinical use. SCFA derivatives can obviate these limitations. The butyrate derivative BA-NH-NH-BA consists of two butyrate molecules with a -NH-O-NH- linker. It inhibits HDAC activity at a lower level compared to butyrate, and mitigates *S. aureus* growth and the inflammatory response in a mouse model (Traisaeng et al., 2019). Taken together, the topical application of SCFAs have broad effects on the skin microbiome, skin barrier, and related inflammatory factors.

### 4.2. Oral supplementation of SCFAs in inflammatory skin diseases

Gut microbes have the ability to modulate systemic inflammation (Bowe et al., 2014), and imbalances in the gut microbes can manifest as inflammatory skin diseases (O’Neill et al., 2016). The beneficial effects of the gut microbiota on host health are closely associated with SCFAs (Tan et al., 2014). Given the similarities in the gut and skin microbiomes, orally administered SCFAs can potentially exert an anti-inflammatory effect on the skin as well (Schwarz et al., 2017).

Probiotics are living microorganisms that have beneficial effects on host health, and can maintain intestinal homeostasis by restoring SCFA levels (Zanten et al., 2012). Studies increasingly show that skin inflammation is closely related to SCFA production. Oral administration of *Bifidobacterium adolescentis* in mice with DNFB-induced AD significantly improves AD-like symptoms, such as skin damage and swelling, which correlates with increased levels of propionate and butyrate and decreased levels of isovalerate (Fang et al., 2020). Regular cheese consumption also introduces probiotics into the gastrointestinal tract and increases the production of SCFAs (Ríos-Covián et al., 2016; Kim et al., 2019). In one study, the neuroprotective effects of *Lactococcus chungangensis* CAU 28-fermented cream cheese and *L. chungangensis* CAU 28 dry cells are compared in a mouse model of AD. The CAU 28 cream cheese results in better outcomes, which can be attributed to a more abundant supply of SCFAs compared to the bacterial cells. Furthermore, oral administration of CAU 28 cream cheese induces a coordinated immune response involving SCFAs and gut microbiota, and effectively improves symptoms of AD (Kim et al., 2019). Oral supplementation with multistrain probiotics (IRT5) can be the alternative therapeutics for the prevention and treatment of skin allergies. Its mechanism of action is mainly attributed to IRT5-induced propionate which is the key immunomodulatory metabolite for the Treg cells expansion and relieving skin inflammation (Kang H. J. et al., 2021). Moreover, A study has showed that sialyllactose and galactooligosaccharides are capable to promote re-epithelialization and repair epidermal wound, which is related to differential changes in SCFA profiles (Perdijk et al., 2019).

Glycomacropeptides (GMPs) are bioactive peptides extracted from dietary proteins that are beneficial to human health (Artym and Zimecki, 2013). Prophylactic feeding of glycopeptides induces SCFA production, and prevents and reverses AD-like skin lesions in rats. Studies show that the protective effect of GMPs on the skin barrier may be mediated by the direct effects of acetate and butyrate on local skin cells (Jiménez et al., 2020). Taken together, oral administration of SCFAs can alleviate the symptoms of inflammatory skin diseases. More clinical studies are needed to confirm the effectiveness of oral supplementation of SCFAs.

## 5. Conclusion

The skin anti-inflammatory effects of SCFAs have been studied in rodents experimental models, but the implication of these findings to the human population is still debatable. Restoring the production of SCFAs may relieve the symptoms of skin inflammation by directly inhibiting the inflammatory factors, improving immune homeostasis, and preventing colonization by pathogenic bacteria. Nevertheless, the mechanism of SCFAs toxicities remains largely unknown.

The current understanding of SCFAs-associated anti-inflammatory effects in inflammatory skin diseases is still limited and is a nascent area of research that requires further investigation. Targeted metabolomics of lesioned skin specimens in patients with inflammatory dermatoses may provide new insights into the molecular basis of their action. Several studies focus on the predominant SCFAs including acetate, propionate, and butyrate. Nevertheless, little is known regarding the effects of other SCFAs, which may play a key anti-inflammatory role. Future studies on valerate and isobutyrate may bring new perspectives on the effects of SCFAs on inflammatory skin diseases. Given the unpleasant taste of SCFAs, it is necessary to develop derivatives that are functionally similar and can be incorporated into clinical application. However, the long-term safety and efficacy of SCFA derivatives warrant further investigation. Encouraging evidence supports the effect of SCFAs in the treatment of AD, psoriasis, acne, and urticaria and SCFAs have therapeutic potential against chronic wounds, alopecia, and seborrheic dermatitis as well. Given the close correlation between SCFA expression and skin inflammation occurrence, SCFAs could be used to detect the therapeutic efficacy and to predict the prognosis of inflammatory skin diseases. A deeper understanding of SCFAs in inflammatory skin diseases provides a systematic theoretical basis for studying SCFAs as potential drugs for promoting human skin health.

## Author contributions

XX, XH, and JY: conceptualization and supervision. WC, ZZ, LW, HQ, DZ, and PX: resources. XX, XH, JY, ZZ, YXL, and LW: literature review and writing – original draft preparation. XX, XH, RJ, YL, YS, and JL: writing – review and editing. All authors have read and agreed to the published version of the manuscript.
